# Volatile aroma profiles and associated microbiota of Yunnan Shiping sour-pulp-fermented stinky tofu

**DOI:** 10.1016/j.fochx.2026.104217

**Published:** 2026-07-14

**Authors:** Yanhui Xu, Lanfeng Ji, Ting Liu, Lvjun Qian, Haoyu Zhou, Canying Zhuang, Hao Chen, Xiaohu Zhou, Liangzhong Zhao, Xiaojie Zhou

**Affiliations:** a College of Food and Chemical Engineering, Shaoyang University, Shaoyang, China; b 3Hunan Provincial Key Laboratory of Soybean Products Processing and Safety Control, Shaoyang, China; c College of Preschool Education and Health Care, Xiangzhong Normal College For Preschool Education, Shaoyang, China; d Industry Research Institute of Shiping Tofu, Shiping, China

**Keywords:** Stinky tofu, Sour-pulp fermentation, Volatile aroma compounds, ROAV, Microbial community, Amplicon sequencing

## Abstract

Yunnan Shiping stinky tofu is a traditional sour-pulp-fermented soybean product with distinctive volatile aroma characteristics. In this study, headspace solid-phase microextraction-gas chromatography–mass spectrometry (HS-SPME-GC–MS), relative odor activity value (ROAV) analysis, and 16S rRNA/ITS amplicon sequencing were used to characterize volatile organic compounds (VOCs) and associated microbial communities in samples collected from four local production facilities. A total of 418 VOCs were detected, and ROAV analysis indicated that sulfur-containing compounds, heterocyclic compounds, furanone derivatives, terpenoid-derived odorants, and unsaturated aldehydes contributed substantially to the volatile aroma profile. The bacterial communities were mainly composed of Proteobacteria, Bacteroidota, and Firmicutes, whereas the fungal communities were dominated by Ascomycota and Basidiomycota. Correlation analysis suggested potential associations between selected bacterial or fungal genera and aroma-active compounds; however, these associations should be interpreted as hypothesis-generating rather than causal evidence. These findings provide a descriptive basis for understanding the volatile aroma characteristics and microbial ecology of Yunnan Shiping sour-pulp-fermented stinky tofu and may guide future targeted isolation, safety assessment, and validation of aroma-associated microorganisms.

## 1 Introduction

Fermented soybean products are widely consumed in many regions of the world and are valued for their distinctive sensory properties, nutritional value, and potential health benefits (Cuamatzin-García et al., 2022). As indicated by Tamang et al. (Tamang et al., 2016), the development of complex aroma profiles in products such as soy sauce, fermented bean paste, natto, and fermented tofu is closely related to microbial fermentation (Harahap et al., 2025). Among these products, stinky tofu is a representative traditional Chinese fermented soybean food, widely recognized for its distinctive aroma characteristics (Dai et al., 2023; Gu et al., 2018). The volatile aroma of stinky tofu is mainly associated with volatile organic compounds (VOCs), including alcohols, aldehydes, ketones, esters, phenols, sulfur-containing compounds, and heterocyclic compounds (Luo et al., 2025). These compounds may be generated from microbial transformation of proteins, lipids, carbohydrates, and amino-acid-derived intermediates. Proteases, lipases, deaminases, decarboxylases, esterases, and other microbial enzymes can release or transform peptides, free amino acids, free fatty acids, organic acids, alcohols, aldehydes, and esters, thereby contributing to aroma development during fermentation (An et al., 2023; Tang et al., 2022; Zhang et al., 2024). Therefore, describing the relationships between microbial communities and volatile compounds is useful for generating hypotheses about aroma formation in fermented soybean products.

Yunnan Shiping stinky tofu is a distinctive regional variety of naturally fermented tofu produced in Yunnan Province, China (Ma & Wu, 2025). In contrast to brine-fermented stinky tofu products, such as Changsha stinky tofu, Yunnan Shiping stinky tofu is produced using traditional sour pulp (acid slurry), which functions as both a natural coagulant and a source of indigenous microorganisms (J. Zhao et al., 2025). The sour pulp is typically derived from fermented soybean whey, and the subsequent spontaneous fermentation under local production conditions may shape both microbial community assembly and VOC formation (Liu et al., 2022; Xu et al., 2019). Compared with brine-based or inoculated fermentation systems, the sour-pulp process may produce a distinct microbial-ecological background; however, the taxa detected at the genus level should be interpreted as characteristic or aroma-associated taxa in this study rather than as confirmed unique or functional microorganisms without further strain-level validation.

The objective of this study was to characterize the volatile aroma profiles and microbial community structures of Yunnan Shiping stinky tofu collected from four local production facilities. GC–MS was employed to identify VOCs, ROAV analysis was used to estimate aroma-active contributors, and high-throughput sequencing was used to analyze bacterial and fungal communities. Multivariate and correlation analyses were further applied to explore potential associations between microbial taxa and volatile aroma compounds. Because the study design was observational and correlation-based, the results are interpreted as descriptive evidence and hypothesis-generating associations, rather than direct proof of causal fermentation mechanisms or starter-culture functionality.

## 2 Materials and methods

### 2.1 The processing and collection of Yunnan stinky tofu sample

**Fig. 1:**
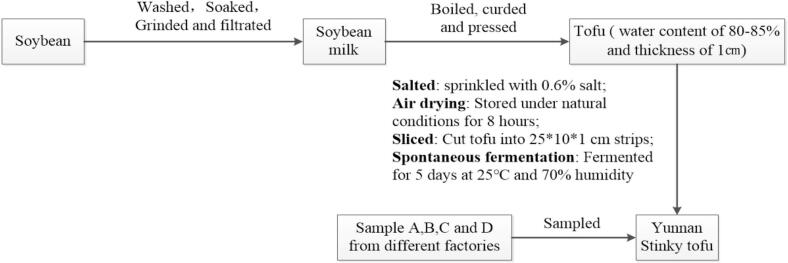
The processing and sampling of Yunnan Shiping stinky tofu.

The processing procedure of Yunnan Shiping stinky tofu is illustrated in Fig. 1. The soybeans were initially washed and soaked in clean water, followed by grinding and filtration to produce soybean milk. Subsequently, the soybean milk was subjected to boiling and coagulation, resulting in the formation of a tofu curd. The curd was subjected to pressurization in order to extract excess water, thereby yielding tofu blocks with a moisture content ranging from 80 to 85% and a thickness of approximately 1 cm. The tofu blocks were subsequently treated with a solution of 0.6% (*w*/w) NaCl, following which they were dried under ambient conditions for a period of eight hours. Thereafter, the salted tofu was fragmented into small pieces and subjected to spontaneous fermentation at a temperature of 25 °C and a relative humidity of 70% for a duration of five days.

Samples of stinky tofu were collected from four local production facilities in Yunnan Shiping and were designated as SP-A, SP-B, SP-C, and SP-D. The four facilities were selected to represent inter-factory variation within the same regional sour-pulp-fermented product category. All samples were collected at the edible fermented stage according to the local production process shown in Fig. 1. After collection, the samples were weighed, immediately frozen in liquid nitrogen, transported under frozen conditions, and stored at −80 °C until further analysis. Because the factories were not experimentally controlled for a single processing variable, the grouping in this study was used to describe production-site-associated variation rather than to infer the effect of any individual factor such as raw material, sour-pulp microbiota, fermentation environment, or production scale.

A total of 12 fermented-tofu samples were included in the study, with three separately labelled samples per production facility (SP-A1 to SP-A3, SP-B1 to SP-B3, SP-C1 to SP-C3, and SP-D1 to SP-D3). The 12 samples were processed and analyzed separately; the three samples within each facility group were treated as biological replicates for VOC and amplicon-sequencing analyses. This cross-sectional field survey was designed to describe within- and between-facility variation, not to estimate a distinct production-batch or collection-date effect.

### 2.2 Microbial detection and analysis

The method of Wang et al. (Wang et al., 2018) was followed with slight modifications. Genomic DNA extracted from the samples via the SDS method was diluted to 1 ng/μl and used as templates to amplify the bacterial 16S rRNA V4 region and fungal ITS1 region using primer pairs 515F/806R and ITS5-1737F/ITS2-2043R, respectively. After amplicon purification, sequencing libraries were constructed using the TruSeq® DNA PCR-Free Sample Preparation Kit (Illumina, San Diego, CA, USA). The libraries were then sequenced on an Illumina NovaSeq 6000 platform. The raw paired-end reads that were generated were subsequently merged using FLASH (v1.2.7), and then quality-filtered via QIIME (v1.9.1). Finally, the chimeric reads were removed by VSEARCH, resulting in the generation of effective tags. Subsequently, these tags were clustered into Operational Taxonomic Units (OTUs) at a 97% similarity threshold utilizing the UPARSE algorithm (v7.0.1001). Taxonomic annotation of representative OTU sequences was conducted using the BLAST method within QIIME against reference databases, including UNITE (v8.2) for fungi. Finally, multiple sequence alignments were executed using MUSCLE (v3.8.31) to facilitate downstream evaluations, and alpha diversity indices (Observed species, Chao1, Shannon, Simpson, ACE, and Good's coverage) were calculated employing QIIME.

After quality filtering and chimera removal, the archived 16S rRNA dataset retained 72,789–85,337 effective tags per sample, and the ITS dataset retained 59,595–81,584 effective tags per sample. Rarefaction outputs were examined to assess sequencing-depth adequacy. OTUs entering alpha-diversity, beta-diversity, and correlation analyses were obtained after the stated quality-control, chimera-removal, and 97% similarity-clustering workflow.

### 2.3 Analysis of volatile flavor compounds by GC–MS

The volatile substances were analyzed according to the method of Kang et al. (Kang et al., 2024) with slight modifications. The volatile organic compounds (VOCs) were extracted and analyzed using a method that involved headspace solid-phase microextraction coupled with gas chromatography–mass spectrometry (GC–MS). In summary, approximately 1 g of liquid nitrogen-ground sample was transferred into a 20 ml headspace vial (Agilent, Palo Alto, CA, USA) containing saturated NaCl solution. After a five-minute equilibration at 60 °C, a 120 μm DVB/CAR/PDMS fiber (Agilent) was exposed to the sample headspace for a period of 15 min at the aforementioned temperature. The extracted volatile organic compounds (VOCs) were thermally desorbed at 250 °C for five minutes in splitless mode into an Agilent 8890 gas chromatograph (GC) coupled with a 7000D mass spectrometer. The desorbed compounds were subsequently separated on a DB-5MS capillary column (30 m × 0.25 mm × 0.25 μm) using helium as the carrier gas at a constant flow of 1.2 ml per minute. The initial oven temperature program was set at 40 °C, where it remained for a duration of 3.5 min. Thereafter, the temperature was increased to 100 °C at a rate of 10 °C per minute. The program then proceeded to 180 °C at a rate of 7 °C per minute, followed by an elevation to 280 °C at a rate of 25 °C per minute. This final stage was maintained for a period of 5 min. Mass spectra were acquired in electron impact (EI) ionization mode (70 eV) using selected ion monitoring (SIM), with the ion source, quadrupole, and transfer line temperatures maintained at 230 °C, 150 °C, and 280 °C, respectively.

### 2.4 Estimation of relative odor activity values

ROAV was estimated separately for each production-site group as ROAV_i = (A_i/T_i)/(A_max/T_max) x 100, where A_i is the relative peak-area percentage of compound i in that group, T_i is its reported odor threshold, and A_max/T_max is the largest ratio among the compounds considered in that group. Thus, the values are dimensionless relative estimates based on semi-quantitative peak-area data, not absolute odor activity values derived from quantified concentrations. Odor thresholds were used only as reference values for this relative ranking.

For each reported VOC feature, the archived feature table records a quantitative ion, qualitative ion, and NIST-RI field. Accordingly, compound names in this study are treated as putative library/retention-index annotations rather than comprehensive authentic-standard confirmations. The present work did not use absolute quantification for all reported VOCs; moreover, blank-control metadata and feature-specific library-match cutoffs were not available for verification from the archived analytical record.

### 2.5 Data analyses

The 12 individual samples (*n* = 3 per production-site group) were used for VOC and amplicon-sequencing analyses. For abundance data with three within-group biological replicates, results are expressed as mean ± standard deviation (SD), and one-way analysis of variance (ANOVA) followed by Duncan's multiple-range test was conducted in SPSS 19.0 (IBM Corp., Armonk, NY, USA) at *P* < 0.05. PCA was used to visualize overall variation in VOC profiles. Spearman correlations between selected microbial genera and selected VOCs were calculated across all 12 samples as exploratory association analyses. The displayed correlation maps show correlation coefficients; no correlation was interpreted as causal or as a multiple-testing-corrected inferential finding. Bioinformatics analyses of the high-throughput sequencing data were processed using QIIME and R software packages. The 16S rRNA and ITS amplicon sequencing data generated in this study have been deposited in the National Center for Biotechnology Information (NCBI) Sequence Read Archive (SRA) under accession number PRJNA1152245.

## 3 Results and discussion

### 3.1 Volatile organic compound (VOC) profiles of Yunnan stinky tofu

#### 3.1.1 Overall volatile flavor profile of Yunnan stinky tofu

**Fig. 2:**
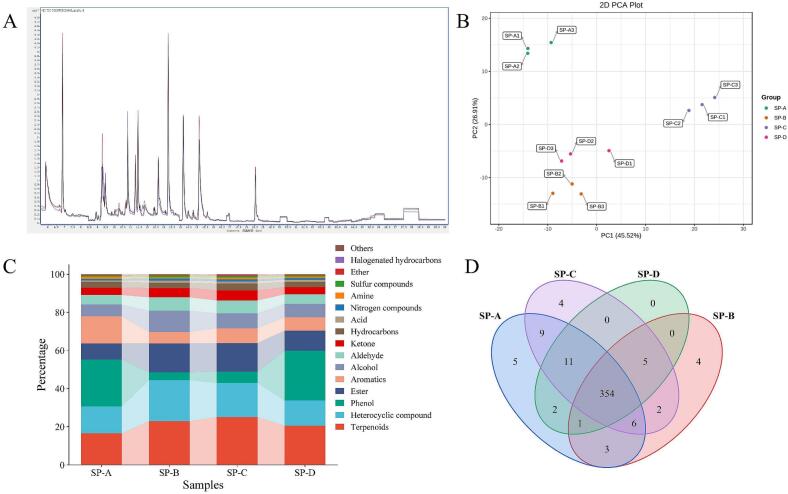
Volatile flavor profiles of Yunnan stinky tofu samples from different factories. (A) Total ion chromatograms (TIC) of volatile compounds detected by GC–MS. (B) PCA score plot showing the discrimination of samples (SP-A, SP-B, SP-C, and SP-D) based on volatile compounds. (C) Relative abundance of different classes of volatile compounds. (D) Venn diagram showing shared and unique volatile compounds among the four samples.

The volatile flavor profiles of Yunnan stinky tofu from four different factories were systematically characterized using GC–MS. As shown in the total ion chromatograms (TIC, Fig. 2A), all samples exhibited complex and diverse volatile profiles. According to their chemical structures, the identified volatile organic compounds (VOCs) were classified into several groups, including esters, alcohols, aldehydes, ketones, hydrocarbons, acids, sulfur compounds, phenols, aromatics, and heterocyclic compounds (Fig. 2C). Among these compounds, heterocyclic compounds, phenols, alcohols, and esters were the dominant volatile classes in all samples. These compounds are widely recognized as key contributors to the aroma of fermented soybean products and are mainly generated through microbial metabolism of amino acids, carbohydrates, and phenolic precursors during fermentation (Q. Li et al., 2023; Qiu et al., 2022). Although the overall volatile composition was similar among samples, notable differences in the relative abundance of certain chemical classes were observed. For example, SP-D contained a higher proportion of phenolic compounds, whereas SP-B and SP-C showed higher levels of heterocyclic compounds and alcohols, suggesting differences in microbial metabolic activities and fermentation conditions among factories (Wen et al., 2024).

To further investigate the distribution of volatile compounds, a Venn diagram was constructed (Fig. 2D). A total of 354 VOCs were shared among all four groups, representing the core volatile profile of Yunnan sour-pulp stinky tofu. In addition, several unique compounds were detected in individual groups, with 5, 4, 4, and 3 VOCs uniquely identified in SP-A, SP-B, SP-C, and SP-D, respectively. These results indicate that while a common flavor foundation exists across samples, variations in processing conditions and microbial communities may contribute to the formation of specific volatile compounds (M. Zhao et al., 2025).

Principal component analysis (PCA) was further performed to evaluate the overall differences in volatile profiles among samples (Fig. 2B). The first two principal components explained 72.43% of the total variance (PC1 = 45.52%, PC2 = 26.91%), indicating that PCA effectively captured the major variations in volatile composition. The score plot showed clear separation among the four sample groups. SP-A and SP-B were distributed on the left side of the PC1 axis but separated along PC2, whereas SP-C and SP-D were located on the right and lower regions of the plot. This distinct clustering pattern suggests that although sour-pulp fermentation produces a broadly similar product type, differences in environmental conditions and fermentation microecosystems among factories lead to distinct volatile flavor characteristics (Sun et al., 2025).

#### 3.1.2 Differential volatile compounds and key aroma contributors in Yunnan stinky tofu

Table 1 summarizes the top 30 abundance-dominant volatile compounds across the four production-site groups. Phenol was the most prevalent volatile compound in SP-A (23.50%) and SP-D (24.59%). Phenolic compounds are frequently produced through the microbial degradation of aromatic amino acids, such as tyrosine. These compounds are known to contribute aromas that are frequently described as smoky, pungent, and medicinal to fermented soybean products (Liang et al., 2024). Furthermore, heterocyclic compounds, such as furans and pyrazoles, were detected in all samples. These compounds are frequently produced through Maillard reactions and microbial metabolism, often contributing to roasted and nutty flavor notes (Sun et al., 2026).

**Table 1:** The top 30 volatile flavor substances detected in four stinky tofu samples.

**Compounds**	**Class**	**CAS**	**Relative percentage (%)**			
			**SP-A**	**SP-B**	**SP-C**	**SP-D**
Phenol	Phenol	108–95-2	23.5 ± 1.18a	2.34 ± 0.12c	4.23 ± 0.83b	24.59 ± 1.20a
Phenol, 4-propyl-	Phenol	645–56-7	1.01 ± 0.04c	1.58 ± 0.05a	1.57 ± 0.03a	1.23 ± 0.05b
Carvenone	Terpenoids	499–74-1	2.24 ± 0.06c	3.53 ± 0.13a	3.51 ± 0.04a	2.68 ± 0.06b
6-Octen-1-ol, 7-methyl-3-methylene-	Terpenoids	13,066–51-8	1.08 ± 0.03c	1.64 ± 0.03a	1.62 ± 0.08a	1.25 ± 0.06b
*endo*-Borneol	Terpenoids	507–70-0	0.72 ± 0.02d	1.21 ± 0.08b	1.95 ± 0.11a	0.94 ± 0.11c
1,5-Heptadien-4-ol, 3,3,6-trimethyl-	Terpenoids	27,644–04-8	1.03 ± 0.11b	1.43 ± 0.09a	1.23 ± 0.02b	1.13 ± 0.11b
2-Hexanol, (*R*)-	Alcohol	26,549–24-6	1.33 ± 0.04b	1.52 ± 0.17a	1.13 ± 0.03c	1.07 ± 0.08c
Benzenemethanol, alpha-2-propenyl-	Alcohol	936–58-3	0.89 ± 0.06c	1.39 ± 0.03a	1.47 ± 0.03a	1.11 ± 0.05b
1-Octen-3-ol	Alcohol	3391-86-4	0.71 ± 0.04c	2.25 ± 0.09a	0.76 ± 0.05c	1.00 ± 0.03b
2-Hepten-1-ol, (*E*)-	Alcohol	33,467–76-4	0.70 ± 0.03c	2.20 ± 0.10a	0.73 ± 0.04c	0.99 ± 0.04b
ParAldehyde	Aldehyde	123–63-7	1.38 ± 0.03b	1.58 ± 0.07a	1.10 ± 0.03c	1.06 ± 0.08c
1-Cyclohexene-1-carboxAldehyde,4-(1-methylethenyl)-, (*S*)-	Aldehyde	18,031–40-8	0.84 ± 0.02c	1.26 ± 0.02a	1.28 ± 0.02a	0.97 ± 0.06b
Naphthalene, 1,2,3,4-tetrahydro-2,6-dimethyl-	Aromatics	7524-63–2	7.39 ± 0.85a	ND	0.43 ± 0.03c	1.54 ± 0.23b
Benzene, 1,2,4-trimethyl-	Aromatics	95–63-6	0.87 ± 0.03a	0.91 ± 0.02a	0.86 ± 0.01a	0.77 ± 0.05b
(5-bromopentyl)-Benzene	Aromatics	14,469–83-1	0.58 ± 0.04b	0.81 ± 0.10a	0.89 ± 0.06a	0.88 ± 0.09a
(3-Bromo-1-methylpropoxymethyl) benzene	Aromatics	51,666–29-6	0.57 ± 0.04b	0.81 ± 0.08a	0.89 ± 0.08a	0.87 ± 0.08a
Benzene, (1-nitropropyl)-	Aromatics	5279-14-1	2.10 ± 0.16a	0.02 ± 0.00c	0.15 ± 0.01c	0.46 ± 0.07b
2-Butenoic acid, 3-hexenyl ester, (E, Z)-	Ester	65,405–80-3	1.08 ± 0.04c	1.64 ± 0.03a	1.64 ± 0.04a	1.25 ± 0.06b
Benzenepropanoic acid, methyl ester	Ester	103–25-3	0.02 ± 0.01c	1.17 ± 0.08b	2.17 ± 0.16a	1.14 ± 0.12b
4-Penten-1-ol, propanoate	Ester	30,563–30-5	0.67 ± 0.04c	2.21 ± 0.10a	0.74 ± 0.05c	0.97 ± 0.03b
Isobutyl acetate	Ester	110–19-0	1.10 ± 0.09b	1.35 ± 0.09a	0.93 ± 0.04c	0.87 ± 0.10c
Benzyl tiglate	Ester	37,526–88-8	0.57 ± 0.04b	0.80 ± 0.09a	0.88 ± 0.07a	0.88 ± 0.08a
2-Butenoic acid, 2-methyl-, 2-methylpropyl ester	Ester	66,917–61-1	0.61 ± 0.02c	0.91 ± 0.02a	0.89 ± 0.01a	0.71 ± 0.03b
Furaneol	Heterocyclic compound	3658-77-3	3.14 ± 0.08a	3.21 ± 0.09a	2.60 ± 0.14b	1.78 ± 0.07c
2-Methyl-3-furanthiol	Heterocyclic compound	28,588–74-1	1.61 ± 0.11c	2.57 ± 0.09a	2.54 ± 0.06a	2.11 ± 0.18b
Furan, 2-pentyl-	Heterocyclic compound	3777-69-3	1.87 ± 0.03b	4.82 ± 0.17a	1.14 ± 0.09c	1.00 ± 0.09c
1H-Pyrazole-1-carboximidamide, 3,5-dimethyl-	Heterocyclic compound	22,906–75-8	1.46 ± 0.04c	2.17 ± 0.06a	2.18 ± 0.04a	1.75 ± 0.07b
2(5H)-Furanone, 5-ethyl-3-hydroxy-4-methyl-	Heterocyclic compound	698–10-2	1.08 ± 0.03b	1.59 ± 0.08a	1.65 ± 0.05a	1.18 ± 0.09b
Ethanone, 1-(1H-pyrazol-4-yl)-	Heterocyclic compound	25,016–16-4	0.59 ± 0.01d	1.03 ± 0.05b	1.73 ± 0.11a	0.79 ± 0.10c
2-Acetyl-4,4-dimethyl-cyclopent-2-enone	Ketone	81,979–96-6	1.03 ± 0.03c	1.55 ± 0.03a	1.52 ± 0.04a	1.18 ± 0.05b

Note: ”ND” denotes not detected;

While relative abundance reflects the semi-quantitative distribution of volatile compounds, the actual contribution of each compound to aroma perception is strongly influenced by its odor threshold (OT). Consequently, the relative odor activity value (ROAV) was calculated to estimate aroma-active contributors in Yunnan stinky tofu (Table 2). Compounds with an ROAV greater than 1 are generally considered important contributors to the perceived aroma profile (Zhang et al., 2022). Accordingly, Table 1 should be interpreted as an abundance-based summary, whereas Table 2 identifies compounds with estimated odor activity. Several aldehydes were therefore not among the top 30 most abundant VOCs but still had ROAV values greater than 1 because of their low odor thresholds.

**Table 2:** Estimated relative odor activity values (ROAVs) of selected VOCs.

**Compounds**	**Description**	**OT (mg/kg)**	**SP-A**	**SP-B**	**SP-C**	**SP-D**
Isobutyl acetate	Fruity, sweet	0.0080000	0.06	0.05	0.03	0.04
2(5H)-Furanone, 5-ethyl-3-hydroxy-4-methyl-	Sweet	0.0000240	18.50	18.94	19.06	16.39
Furan, 2-pentyl-	Green, beany	0.0048000	0.16	0.29	0.07	0.07
Furaneol	Caramel, swee	0.0100000	0.13	0.09	0.07	0.06
2-Methyl-3-furanthiol	Meaty, roasted	0.0048100	0.14	0.15	0.15	0.15
Carvenone	Minty, herbal	0.0220000	0.04	0.05	0.04	0.04
1-Octen-3-ol	Mushroom-like	0.0020000	0.15	0.32	0.11	0.17
2-Nonenal, (*E*)-	Fatty, green	0.0000650	1.51	1.72	2.58	1.49
(Z, *Z*)-3,6-Nonadienal	Green	0.0000500	1.81	1.88	1.88	1.78
6-Nonenal, (Z)-	Fresh, green	0.0000200	7.74	7.63	7.69	7.32
2-Methylisoborneol	Musty, earthy	0.0000022	72.45	73.52	75.49	70.58
2-Thiophenemethanethiol	Roasted, meaty	0.0000010	100	100	100	100
Linalool	Floral, citrus-like	0.0015000	0.11	0.10	0.11	0.10

Note: OT denotes the odor threshold obtained from published threshold compilations and literature. ROAV values are semi-quantitative relative estimates calculated from group-specific relative peak-area percentages and odor thresholds.

Among these compounds, 2-thiophenemethanethiol showed the highest estimated odor contribution (ROAV = 100) across all samples. This sulfur-containing heterocyclic compound may be related to the transformation of sulfur-containing amino acids such as methionine and cysteine during fermentation and is associated with roasted and meaty aroma notes in fermented foods (Chen et al., 2024). 2-Methylisoborneol contributed substantially to earthy and musty notes, whereas 5-ethyl-3-hydroxy-4-methyl-2(5H)-furanone contributed sweet and caramel-like notes. Unsaturated aldehydes such as (Z)-6-nonenal, (E)-2-nonenal, and (Z,Z)-3,6-nonadienal showed relatively low abundance compared with the top abundance-dominant VOCs, but their low odor thresholds resulted in ROAV values greater than 1. These results indicate that the volatile aroma profile of Yunnan sour-pulp stinky tofu is shaped by both concentration-dominant compounds and low-threshold aroma-active compounds.

### 3.2 Microbial composition analysis based on high-throughput sequencing

#### 3.2.1 Microbial diversity and community distribution of bacteria and fungi in Yunnan stinky tofu

High-throughput sequencing was performed to investigate the microbial diversity and community distribution in Yunnan stinky tofu samples from four different factories. Alpha diversity indices, Venn diagrams, and beta diversity analyses were used to evaluate the microbial diversity and structural differences of both bacterial and fungal communities.

**Fig. 3:**
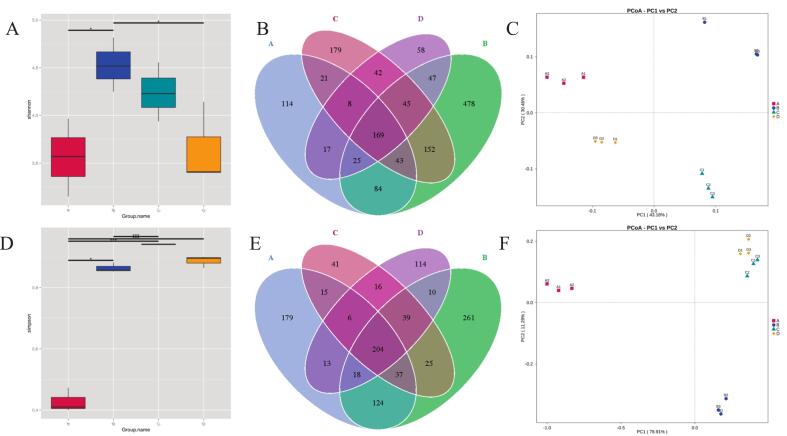
Diversity and community distribution of bacterial and fungal microbiota in Yunnan stinky tofu. (A) Shannon diversity index of bacterial communities. (B) Venn diagram showing shared and unique bacterial OTUs among samples. (C) PCoA plot of bacterial community structure. (D) Simpson diversity index of fungal communities. (E) Venn diagram showing shared and unique fungal OTUs. (F) PCoA plot of fungal community structure.

Alpha diversity analysis was used to evaluate the richness and evenness of microbial communities within each sample. For the bacterial communities (Fig. 3A), the Shannon index demonstrated that samples from SP-B exhibited the highest bacterial diversity, followed by SP-C, SP-D, and SP-A. A similar trend was observed for the Simpson index, indicating that SP-B possessed a more complex and evenly distributed bacterial community. Conversely, the fungal alpha diversity (Fig. 3D) exhibited a divergent pattern, with SP-B and SP-D demonstrating relatively higher Simpson index values, while SP-A exhibited the lowest fungal diversity. These results suggest that spontaneous sour-pulp fermentation is strongly influenced by environmental conditions and fermentation practices, which may lead to differences in microbial community structure among factories (Wei et al., 2024; Zhang et al., 2026).

The distribution of shared and unique operational taxonomic units (OTUs) was further analyzed using Venn diagrams. As demonstrated in Fig. 3B and E, a mere eight bacterial OTUs and six fungal OTUs were identified as shared among all four groups, and were therefore described as shared OTUs detected across all four production-site groups. Conversely, a substantial number of unique OTUs were identified in each group. Specifically, SP-B exhibited 478 unique bacterial OTUs and 261 unique fungal OTUs, while SP-A contained 114 and 179, respectively. The coexistence of a small shared-OTU set and numerous unique OTUs indicates that local environmental conditions, processing practices, and indigenous microorganisms may strongly influence microbial community assembly during fermentation (L. Wang et al., 2025; Xiao et al., 2023).

The Venn-diagram overlap was defined by OTU presence or absence after sequence processing and 97% OTU clustering. No abundance or prevalence threshold was applied to designate a core microbiota. Therefore, the shared OTUs are reported only as OTUs detected across all four production-site groups and should not be interpreted as an abundance-defined core community.

To further examine differences in microbial community composition among samples, principal coordinate analysis (PCoA) based on beta diversity was conducted. The bacterial PCoA plot (Fig. 3C) exhibited a clear separation among the four sample groups, with PC1 and PC2 explaining 43.18% and 30.46% of the total variation, respectively. Conversely, the fungal PCoA plot (Fig. 3F) exhibited distinct clustering of samples, with PC1 accounting for 78.91% of the total variance. In both analyses, biological replicates from the same factory demonstrated a high degree of similarity, suggesting the reproducibility of the sequencing results was satisfactory. These findings indicate that microbial community structures exhibited substantial variation among samples from disparate factories. Such microbial variations may contribute to the differences in volatile flavor profiles observed in Section 3.1 (Xu et al., 2021).

#### 3.2.2 Microbial community composition and differential taxa of bacteria and fungi in Yunnan stinky tofu

**Fig. 4:**
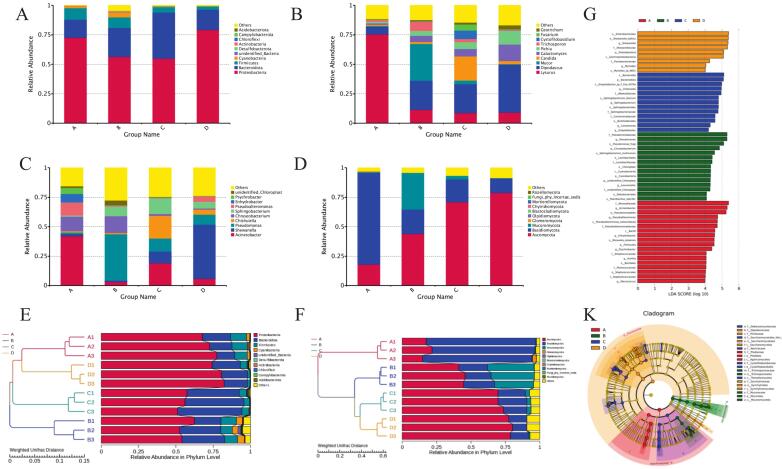
Composition and differential analysis of bacterial and fungal communities in Yunnan stinky tofu. (A) Relative abundance of bacterial communities at the phylum level. (B) Relative abundance of fungal communities at the genus level. (C) Relative abundance of bacterial communities at the genus level. (D) Relative abundance of fungal communities at the phylum level. (E) Cluster analysis of bacterial communities at the phylum level. (F) Cluster analysis of fungal communities at the phylum level. (G) LEfSe analysis showing differential bacterial taxa among samples. (K) Cladogram indicating significantly different microbial taxa.

To further characterize the microbial composition of sour-pulp fermentation, the taxonomic distribution of bacterial and fungal communities was analyzed at multiple taxonomic levels. At the phylum level, the bacterial communities across all samples were predominantly composed of Proteobacteria and *Bacteroidota*, with smaller proportions of *Firmicutes* and *Actinobacteriota* (Fig. 4A). Among these, Proteobacteria were the most prevalent phylum, particularly in SP-D. Members of these phyla are frequently reported as dominant microorganisms in fermented soybean products and are known to participate in the degradation of proteins, lipids, and carbohydrates during fermentation (Y. Li et al., 2023).

Similarly, the composition of the fungal communities was predominantly *Ascomycota* and *Basidiomycota* (Fig. 4D). *Ascomycota* was the predominant fungal phylum in SP-C and SP-D, whereas *Basidiomycota* exhibited comparatively higher abundance in SP-A. These fungal phyla are widely detected in fermented foods and play important roles in carbohydrate metabolism and the formation of aroma compounds (Hong et al., 2026).

At the genus level, substantial differences in microbial composition were observed among the four factory groups (Fig. 4C). The predominant bacterial genera included Acinetobacter, Shewanella, Chryseobacterium, Sphingobacterium, Pseudomonas, and Psychrobacter. Acinetobacter was particularly abundant in SP-A, whereas Pseudomonas and Sphingobacterium showed higher relative abundance in SP-B and SP-D, respectively. Some members of these genera have been reported to participate in protein, lipid, and amino acid transformation in fermented foods, which may provide precursors for volatile aroma formation (Zhao et al., 2026). However, because the present study was based on genus-level amplicon sequencing, these taxa should be regarded as aroma-associated candidates rather than confirmed functional microorganisms.

The fungal communities were dominated by Lysurus, Candida, Cladosporium, and Fusarium (Fig. 4B). Yeasts such as Candida were particularly abundant in SP-C and SP-D. Yeasts in fermented foods may contribute to the formation of higher alcohols, esters, and other aroma compounds through carbohydrate and amino acid metabolism (Belleggia & Osimani, 2023). Hierarchical cluster analysis further revealed distinct grouping patterns among samples (Fig. 3E and Fig. 4F). Samples from the same manufacturing facility clustered closely, suggesting acceptable within-group reproducibility. Meanwhile, SP-A and SP-D formed clusters distinct from the SP-B/SP-C groups, indicating production-site-associated differences in microbial community composition.

To identify microbial taxa that contributed to inter-group differences, linear discriminant analysis effect size (LEfSe) was performed (Fig. 4G and Fig. 4K). The LDA score plot revealed significant differences among bacterial taxa across the groups. For instance, Macrococcus and Staphylococcus were enriched in SP-A, whereas Pseudomonas and Acinetobacter were identified as characteristic taxa in SP-B. Sphingobacterium and Chryseobacterium were enriched in SP-C. These taxa may serve as microbial markers for distinguishing samples from different production sites. However, their functional roles in aroma formation require further validation through strain isolation, metabolic characterization, and controlled fermentation experiments.

### 3.3 Correlation analysis between microbial communities and volatile flavor compounds

In order to explore the relationships between microbial communities and volatile compounds in Yunnan stinky tofu, Spearman correlation analysis was performed in order to evaluate the associations between dominant microbial genera and key aroma-active volatile compounds identified by GC–MS.

The correlation calculations used the 12 individual samples (*n* = 12). The displayed heatmaps are exploratory visualizations of Spearman correlation coefficients for selected genera and selected VOCs. No edge-selection threshold, *P*-value cutoff, or multiple-testing correction was used to support confirmatory claims; accordingly, the correlations are discussed only as hypothesis-generating associations.

**Fig. 5:**
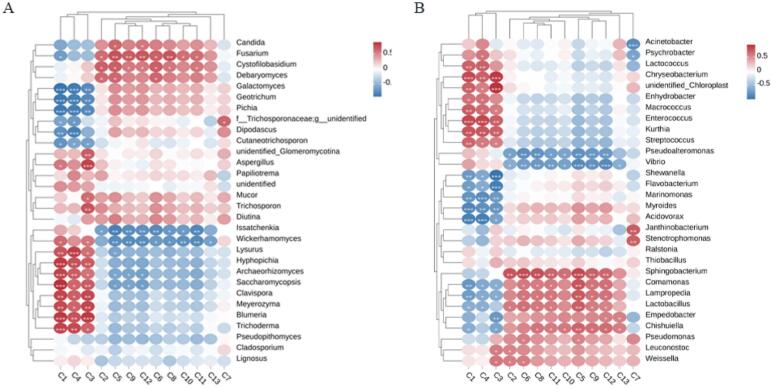
Correlation analysis between microorganisms and volatile flavor compounds in Yunnan stinky tofu. (A) Spearman correlation heatmap between dominant fungal genera and volatile compounds. (B) Spearman correlation heatmap between dominant bacterial genera and volatile compounds. Red and blue colors indicate positive and negative correlations, respectively. (For interpretation of the references to colour in this figure legend, the reader is referred to the web version of this article.)

For the fungal correlation network (Fig. 5A), several yeasts and filamentous fungi showed associations with multiple volatile compounds. Candida, Debaryomyces, Pichia, Geotrichum, and Galactomyces were positively correlated with several alcohols and esters. Based on previous studies of fermented foods, such associations may be related to carbohydrate metabolism, alcohol formation, and esterification, which can contribute to fruity and floral aroma notes (Feixia et al., 2024; S. Wang et al., 2025). Conversely, Wickerhamomyces, Saccharomycopsis, and Trichoderma showed negative correlations with multiple VOCs. These negative correlations may reflect differences in ecological niche, fermentation stage, substrate preference, or co-occurrence structure, rather than direct evidence of substrate competition or metabolite consumption.

The bacterial correlation network (Fig. 5B) further revealed associations between selected bacterial genera and aroma-active compounds. Sphingobacterium and Pseudomonas were positively correlated with sulfur-containing and heterocyclic compounds, including 2-thiophenemethanethiol and 2-methylisoborneol, which were identified as aroma-active compounds based on ROAV analysis. A plausible explanation is that proteolysis and amino acid catabolism may release sulfur-containing amino acids and other precursors that participate in the formation of sulfur-containing and heterocyclic volatiles. Nevertheless, these correlations do not demonstrate that the detected genera directly synthesized these compounds.

Lactic acid bacteria (LAB), including Lactobacillus, Weissella, and Leuconostoc, were positively correlated with several organic acids, ketones, and esters. LAB can acidify the fermentation environment and provide organic-acid-related substrates that may participate in subsequent ester formation (Qiao et al., 2022). In contrast, genera such as Acinetobacter and Macrococcus were negatively correlated with certain heterocyclic compounds. This pattern may indicate that these bacteria are more closely associated with early substrate transformation or with microbial community structures different from those favoring the accumulation of these VOCs. Further functional verification is required to confirm these interpretations.

Overall, the correlation analysis suggests potential links between microbial community composition and volatile metabolites during stinky tofu fermentation. Bacterial genera such as Sphingobacterium, Pseudomonas, and LAB may be associated with protein degradation, amino acid metabolism, and organic acid formation, whereas fungal genera such as Candida and Pichia may be associated with alcohol and ester formation. These results provide hypotheses for future studies on microbial contributions to aroma formation, but they should not be interpreted as direct evidence of causal microbial functions.

From a food application perspective, the taxa identified in this study should be interpreted cautiously. Amplicon sequencing provides genus-level community information but does not determine strain identity, safety status, or technological suitability. Some genera detected in fermented foods may include both useful and undesirable members. Therefore, any future use of aroma-associated taxa as starter cultures would require strain-level isolation and identification, genome-based safety assessment, screening for virulence, toxin, antibiotic-resistance, or undesirable metabolite risks, and validation through inoculated fermentation trials.

## 4 Conclusion

The present study characterized the volatile aroma profiles and microbial community structures of Yunnan Shiping sour-pulp-fermented stinky tofu collected from four local production facilities. GC–MS analysis detected a complex VOC profile comprising esters, alcohols, aldehydes, ketones, acids, heterocyclic compounds, phenols, sulfur-containing compounds, and other chemical classes. Multivariate analysis showed that the four production-site groups had distinguishable volatile profiles. ROAV analysis further identified several aroma-active compounds estimated to contribute to the characteristic volatile aroma profile, including sulfur-containing compounds, heterocyclic compounds, furanone derivatives, terpenoid-derived odorants, and unsaturated aldehydes.

Amplicon sequencing revealed diverse bacterial and fungal communities in the fermentation system. The bacterial communities were predominantly composed of Proteobacteria, Bacteroidota, and Firmicutes, whereas the fungal communities were mainly composed of Ascomycota and Basidiomycota. At the genus level, Acinetobacter, Pseudomonas, Sphingobacterium, Lactobacillus, Weissella, Candida, and Debaryomyces were among the dominant or characteristic taxa. The observed differences among the four production sites suggest that local production conditions may be associated with microbial community variation, although the present design does not allow individual processing factors to be separated.

Correlation analysis revealed associations between selected microbial genera and VOCs. These associations suggest potential microbial-metabolite links related to protein degradation, amino acid metabolism, organic acid production, alcohol formation, and esterification. However, because the evidence is based on genus-level sequencing and correlation analysis, the results should be interpreted as hypothesis-generating rather than causal.

In conclusion, this study provides a descriptive profile of volatile aroma compounds and associated microbiota in Yunnan Shiping sour-pulp-fermented stinky tofu. The findings provide a basis for future targeted studies on aroma-associated microorganisms, but strain isolation, safety assessment, quantitative aroma validation, and controlled fermentation experiments are required before the detected taxa can be considered functional starter candidates or before specific microbial mechanisms can be confirmed.

## CRediT authorship contribution statement

**Yanhui Xu:** Writing – original draft, Methodology, Conceptualization. **Lanfeng Ji:** Methodology, Investigation, Funding acquisition. **Ting Liu:** Validation, Methodology. **Lvjun Qian:** Data curation. **Haoyu Zhou:** Methodology, Investigation. **Canying Zhuang:** Writing – review & editing. **Hao Chen:** Project administration. **Xiaohu Zhou:** Writing – review & editing, Funding acquisition. **Liangzhong Zhao:** Resources, Project administration. **Xiaojie Zhou:** Writing – review & editing, Supervision, Resources, Project administration, Funding acquisition, Conceptualization.

## Funding

This work was supported by The Project of Shaoyang Science and Technology (Grant Nos: 2024GZ1001, 2025GZ2017), College Students' Innovation Training Program Project of Hunan Province(Grant number: S202410547026),The Xiaohe Talent Project of Shaoyang(Grant number: 202408).

## Declaration of competing interest

The authors declare that they have no known competing financial interests or personal relationships that could have appeared to influence the work reported in this paper.

## Data Availability

Data will be made available on request.
